# The amount of spermatic cord rotation magnifies the time-related orchidectomy risk in Intravaginal testicular torsion

**DOI:** 10.1590/S1677-5538.IBJU.2016.0166

**Published:** 2016

**Authors:** Aderivaldo Cabral Dias, João Ricardo Alves, Hélio Buson, Paulo Gonçalves de Oliveira

**Affiliations:** 1 Unidade de Urologia, Hospital de Base do Distrito Federal, Brasília, DF, Brasil; 2 Unidade de Cirúrgica Pediátrica do Hospital de Base do Distrito Federal, Brasília, DF, Brasil; 3 Departamento de Cirurgia da Faculdade de Ciências Médicas da Universidade de Brasília, Brasília, DF, Brasil

**Keywords:** Testis, Ischemia, Spermatic Cord Torsion

## Abstract

**Purpose:**

To investigate the roles of age, testicular rotation and time in the surgical outcome of intravaginal testicular torsion (iTT).

**Patients and Methods:**

We retrieved the records of all iTT patients treated in our unit from January 2012 to January 2014. Explanatory variables were: age (years); presentation delay (PrD, time between symptoms and hospitalization); surgical delay (SurgD, time between hospitalization and surgery) and testicular rotation (rotation), with surgical outcome (orchidopexy, orchidectomy) as response variable. Differences in PrD, SurgD, age and rotation by surgical outcome were evaluated non-parametrically. Step-down logistic regression included age, PrD, SurgD and rotation as predictors. Statistical significance and confidence intervals (CI) were set at p<0.05 and 0.95. Odds ratios (OR) were computed from the model's coefficients.

**Results:**

Complete variable information was available for 117 patients, and most (61, 52.1%) underwent orchidectomy. Ages were similar between orchidectomy and orchidopexy patients (median 15.8 vs. 16.0 years, p=0.78). In contrast, PrD (85.0 vs. 8.4 hours, p<0.001), SurgD (3.0 vs. 16.0 hours, p<0.001) were different between orchidectomy and orchidopexy patients. SurgD was similar with PrD<24 hours (4.0 vs. 2.8, p=0.1). Orchidectomy patients had greater rotation (3.0π vs. 2.0π radians, p<0.001). Logistic regression revealed that PrD (OR 0.94; 0.92–0.97; p<0.001) and rotation (OR 0.43; 0.27–0.70; p<0.001) were inversely associated with orchidopexy.

**Conclusion:**

Testicular rotation exerts a multiplicative effect on PrD, so time should not be regarded as the sole predictor of surgical outcome in iTT.

## Introduction

Intravaginal testicular torsion (iTT) is a genuine surgical emergency, affecting mainly adolescents and young adults. After a few hours, ischemic injury leads to testicular necrosis or atrophy. In order to reduce organ loss, the time between the beginning of symptoms and treatment must be reduced.

The importance of swift treatment for iTT has been recognized for more than a century. Nash, in 1893, was able to salvage a testicle by timely intervention (1). Three decades later, Thorek (2) noted that, in both of his cases, the organ would have been saved had immediate surgery been undertaken. Fortunately, testicular salvage rates have been improving, although not everywhere (3, 4). Moreover, it is not unusual to see testicular necrosis with short duration of symptoms, suggesting that the outcome of iTT is not exclusively governed by the duration of ischemia.

One candidate risk factor is the testicular rotation, i.e., the number of turns of the spermatic cord, that appears to damage the organ in a dose-dependent manner: more turns, more vascular occlusion, more ischemia (5, 6). Age is also cited as another risk factor, but reports diverge as to which ages are at increased risk. A worse prognosis associated with younger ages has been attributed to difficulties in communicating symptoms, and to reliance in potentially misleading clinical diagnosis (7–9). On the other hand, older patients were reported to have worse outcomes by holding up to seek medical attention, as well as to diagnostic delays due to the perception that iTT is rare in older adults (10–12).

We found ourselves in a favorable position to investigate the influence of these additional risk factors on the outcome of iTT. Our case load is maximized by the referral of all patients suspected of iTT in our region, a result of the continuous presence of an urologist in our emergency department. Hence, we expect to, with the use of multivariate logistic regression, understand the relationship between ischemia time, testicular rotation and age in the surgical outcome of iTT.

## Patients and Methods

### 

#### Data collection and definition of variables

Following institutional review board approval, we retrieved the medical records of all patients surgically treated for iTT from January 1, 2012 to January 31, 2014. Recovered data included: the patient's ages; date and time of symptoms, as well as date and time of urological examination and operation, as date-time variables; the number of turns (rotation) taken by the testicle, as quantified by the surgeon at operation, in π radians (1.0π radian=180°); and surgical outcome (binary: orchidectomy or orchidopexy). The decision to remove the organ was taken by the surgeon if, after surgical detorsion and placement of the organ at a saline bath at 37°C for at least 20 minutes, no bright red arterial parenchymal bleeding was seen following a stab incision of the tunica albuginea. Only intravaginal torsion cases were included.

Presentation delay (PrD) was calculated subtracting date and time of surgery from date and time of symptom's onset. Surgical waiting times, or surgical delay (SurgD) was calculated as the difference between date and time of hospitalization and date and time of surgery. Treatment delay (TrD) equaled the arithmetic sum of presentation and surgical delays. All variables were included in an electronic spreadsheet for statistical analysis.

### Statistical analysis

We first tested for differences in the explanatory variables according to surgical outcome: orchidectomy or orchidopexy. The second stage of the analysis involved multivariate logistic regression. We also studied SurgD in the subset of patients with PrD less than 24 hours, as, by our unit's treatment algorithm, patients examined within 24 hours are offered immediate surgery, whereas those with later presentation are first examined with Doppler ultrasound, and offered surgery if results indicate iTT. All analysis was conducted with the R Statistical Language, version 3.2.3 (13) and its package rms (14). Statistical significance was established at p<0.05 and confidence intervals (CI) at 0.95.

#### Non-parametric analysis

The explanatory variables age, TrD, PrD, SurgD, and rotation did not distribute normally, so differences were evaluated with the Mann–Whitney test. Also, because of non-normalcy of distributions, the relationship between age and PrD was studied with Kendall's rank-based correlation test.

#### Logistic regression

Backwards step-down logistic regression, removing all cases with missing values, included, initially, the main effects of the predictor variables: Age, PrD, SurgD and rotation, as well as the 2–way interactions: PrD x rotation and SurgD x rotation. We did not include TrD in the initial model, since it is the linear combination of PrD and SurgD. The minimal adequate model was selected with Akaike's Information Criteria (AIC). Probabilities, odds ratios (OR) and relative odds ratios (ROR) were computed from the final model's coefficients. Confidence intervals and statistical significance for model parameters, probabilities, OR and ROR were also established at 0.95 and at p<0.05.

## Results

We identified a total of 142 iTT patients, most of them adolescents (median age 15.8 years, IQR 4.7 years). Median SurgD equaled 5.0 hours, contrasting with a median PrD of 24.5 hours (Table-1). In 25 cases we could not recover information on rotation, so analysis involving this variable was performed on the remaining 117 cases. Overall, 75 patients (52.8%) underwent orchidectomy.

**Table 1:** Overall results.

N = 142	Median	Range	IQR
Age (years)	15.8	8.1 – 42.3	4.7
PrD (hours)	24.5	1.1 – 609.0	77.0
SurgD (hours)	5.4	0.5 – 145.5	18.0
TrD (hours)	30.0	2.83 – 681.0	101.5
N = 117			
Rotation (π radians)	2.0	0.0 - 6.0	1.0

**PrD** = presentation delay; **SurgD** = surgical delay; **TrD** = treatment delay; **Rotation** = testicular rotation; **IQR** = interquartile range

## Bivariate analysis

### 

#### Age

**Figure 1:**
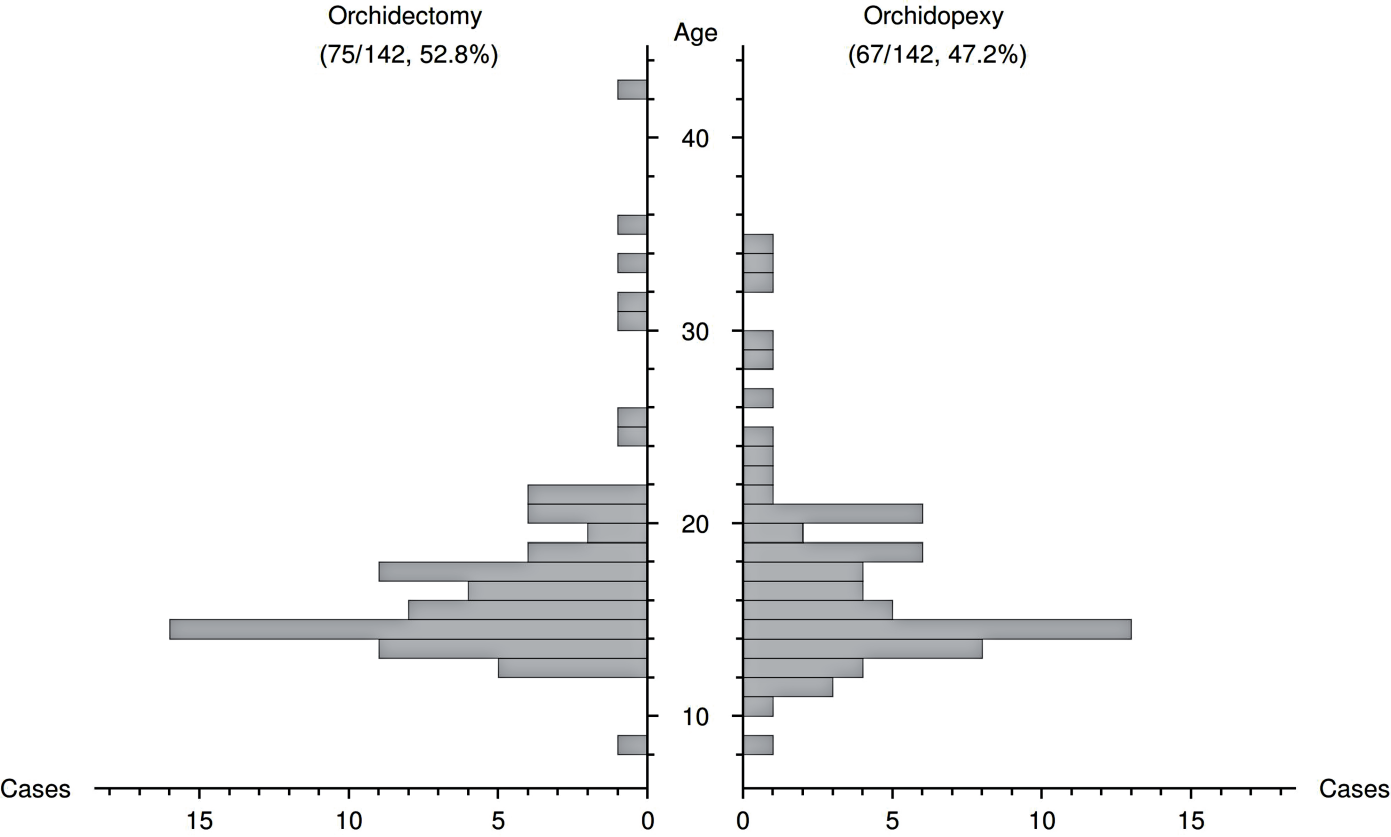
Age distribution of patients either submitted to orchidopexy or orchidectomy.

Age was not significantly different between patients with patients undergoing orchidectomy or orchidopexy (15.7 vs. 15.8 years, p=0.89, Figure-1, Table-2). Also, there was no correlation between age and PrD (Kendall's tau=-0.02, p=0.76).

**Table 2:** Bivariate analysis.

		Overall	Orchidectomy	Orchidopexy	P (MW)
N		142	75 (52,8%)	67 (47,2%)	
Age (years)	Range	8.0 – 42.3	8.0 – 42.3	8.8 – 34.6	0.965
	Median (IQR)	15.8 (4.7)	15.7 (4.6)	15.8 (5.9)	
PrD (hours)	Range	1.1 – 609	3.2 – 609.0	1.1 – 100.0	<0.001
	Median (IQR)	24.5 (77.0)	79.5 (110.8)	8.0 (8.4)	
SurgD (hours)	Range	0.5 – 146.0	0.5 – 146.0	0.5 – 51.0	<0.001
	Median (IQR)	5.3 (18.0)	14.0 (24.5)	2.7 (3.0)	
SurgD with PrD < 24 hours	Range	1.0 – 15.5	1.0 – 15.5	1.0 – 10.5	0.176
	Median (IQR)	2.7 (2.5)	4.0 (2.2)	2.5 (2.5)	
N		117	61	56	
Rotation (π radians)	Range	0.0 – 6.0	1.0 – 6.0	0.0 – 5.0	<0.001
	Median (IQR)	2.0 (1.0)	3.0 (2.0)	2.0 (1.0)	
Age (years)	Range	8.1 – 42.3	8.1 – 42.3	8.9 – 34.6	0.773
	Median (IQR)	15.9 (5.6)	15.8 (4.4)	16.0 (5.8)	
PrD (hours)	Range	1.1 – 609.0	3.2 – 609.0	1.1 – 100.0	<0.001
	Median (IQR)	25.8 (80.9)	85.0 (113.0)	8.4 (10.5)	
SurgD (hours)	Range	0.5 – 114.0	0.5 – 114.0	0.5 – 51.0	<0.001
	Median (IQR)	5.8 (19.3)	16.0 (25.0)	3.0 (3.2)	
SurgD with PrD < 24 hours	Range	1.0 – 10.5	1.0 – 5.7	1.0 – 10.5	0.688
	Median (IQR)	2.9 (2.9)	4.0 (2.0)	2.8 (2.5)	

Upper part of the table with **N** = 142, full dataset; lower part of the table with **N** = 117, dataset with complete variable information.

**IQR** = interquartile range; **PrD** = presentation delay; **SurgD** = surgical delay; **Rotation** = testicular rotation; **MW** = Mann–Whitney's test.

#### Treatment delay

**Figure 2:**
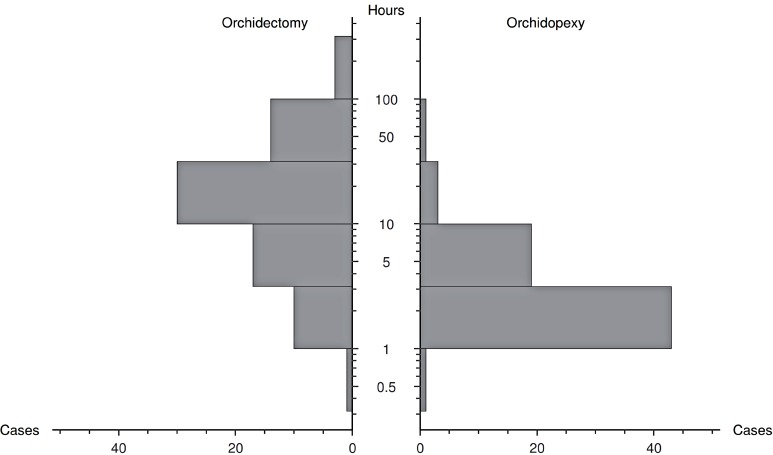
Distribution of presentation delay between patients either submitted to orchidectomy or orchidopexy.

**Figure 3:**
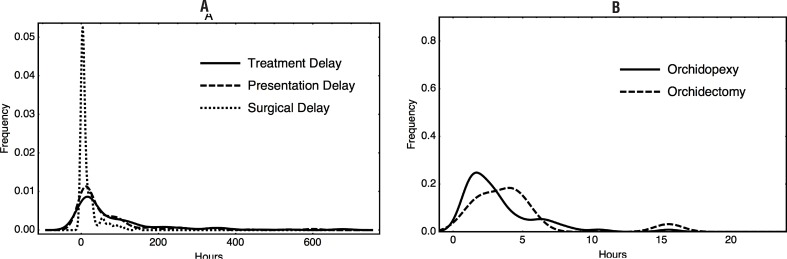
A: Distribution of treatment, presentation and surgical delay among all patients; B: Distribution of surgical delay between patients either submitted to orchidectomy or orchidopexy, with presentation delay < 24 hours

TrD was significantly longer in patients that underwent orchidectomy (median 101.5 vs. 10.5 hours, p < 0.001). Both components of TrD: PrD (median 79.5 vs. 8.0 hours, p < 0.001, Figure-2) and SurgD (median 14.0 vs. 2.7 hours, p < 0.001), were also significantly longer in patients with lost testicles. SurgD was significantly different between patients with PrD shorter or longer than 24 hours (median 2.7 vs 19.0 hours, p < 0.001). However, among patients presenting before 24 hours of symptoms, SurgD was not significantly different between orchidectomy and orchidopexy cases (median 4.0 vs 2.5 hours, p = 0.1; Figure-3). Twelve patients presenting before 24 hours underwent orchidectomy, including a case with PrD shorter than 4 hours. In contrast, 10 patients with PrD longer than 24 hours had their testicles preserved, including a patient symptomatic for 100 hours.

#### Rotation

**Figure 4:**
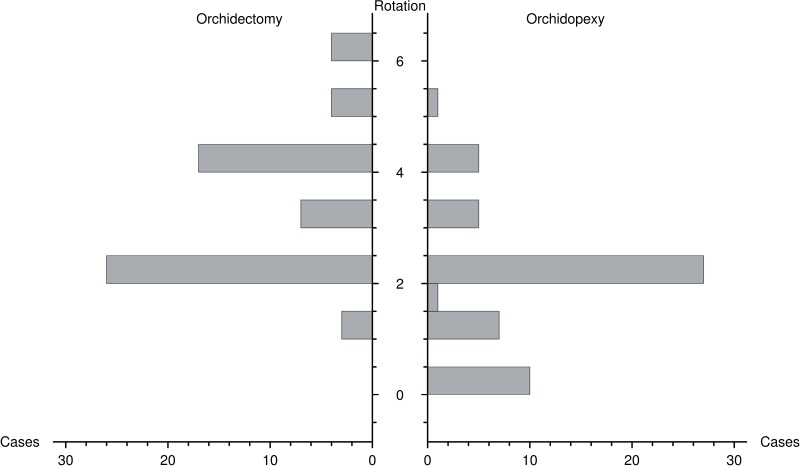
Differences in the magnitude of torsion (Rotation, in π radian) between patients either submitted to orchidectomy or orchidopexy.

Among the 117 patients analyzed, 61 (52.1%) underwent orchidectomy and 56 (47.9%) orchidopexy. Ages, PrD and SurgD had a similar distribution when compared with the full dataset (Table-2), but rotation was significantly different between patients submitted to orchidectomy or orchidopexy (median 3.0π vs. 2.0π radians, p < 0.001, Figure-4). All patients with rotation greater than 5.0π radians underwent orchidectomy, whereas all patients with less than 2.0π radians had their testicles surgically salvaged.

#### Logistic regression

The minimal adequate model included only PrD (z = −4.64, p < 0.001) and rotation (z = −3.41, p < 0.001) as adverse predictors of surgical outcome (Table-3, Figure-4). Rotation decreased the likelihood of orchidopexy after controlling for PrD. The ROR for orchidectomy with an increasing rotation of 1.0π to 2.0π radians, with PrD fixed at 6 hours, equaled 2.30 (CI 1.42 – 3.10), whereas the ROR increasing PrD from 6 to 12 hours with a 2.0π radians rotation was computed at 1.43 (1.23 – 1.66). Table-4 contains the probabilities of orchidopexy according to PrD and rotation, computed from the fitted model.

**Table 3:** Results of multivariate logistic regression.

Parameter	Estimate (CI)	SE	OR (CI)	P
1	4.19 (2.57, 5.82)	0.83	-	<0.001
PrD (hour)	−0.06 (−0.08, −0.03)	0.01	0.94 (0.92, 0.97)	<0.001
Rotation (π radian)	−0.83 (−1.31, −0.35)	0.24	0.43 (0.27, 0.70)	<0.001

**SE** = standard error; **CI95** = 0.95 confidence interval; **OR** = odds ratio for testicular salvage, by increasing the explanatory variables by one unit; **PrD** = presentation delay in hours; rotation, magnitude of rotation, in complete turns.

**Table 4:** Probabilities of testicular salvage, computed from the model.

	PrD (hours)		
Rotation (π radians)	6	12	24
1	0.953 (0.718, 0.995)	0.934 (0.605, 0.994)	0.874 (0.358, 0.990)
2	0.898 (0.450, 0.993)	0.860 (0.331, 0.991)	0.751 (0.152, 0.986)
4	0.624 (0.078, 0.995)	0.538 (0.049, 0.982)	0.363 (0.018, 0.973)

Probability (with 0.95 confidence intervals).

**PrD =** presentation delay; Rotation, magnitude of rotation of the spermatic cord.

## Discussion

We observed an overall low surgical salvage rate (47.2%) with rather long TrD and PrD, reiterating that time is key when treating testicular torsion, for prolonged ischemia times are unequivocally associated with disappointing salvage rates (3, 15, 16). Limited knowledge of this fact may partially explain the 10% salvage rate often seen until 40 years ago (17), and, thankfully, there has been a trend towards better salvage rates in the last decades. Williams (18), in 1979, salvaged 69% of his cases, and Jones (7), in 1986, 75%. The best salvage rate so far recorded, 97.6%, comes from Ireland (19), even though at a cost of a 52.9% false-positive rate. A noteworthy example of diagnostic shrewdness can be seen in the work of Sparks (20), from Rugby, England, presenting a 96% salvage rate as early as 1972.

Delays in treating testicular torsion are sometimes attributed to the lack of diagnostic acumen of emergency department staff, which can lead to medico-legal consequences. However, such argumentation overlooks the fact that treatment delays have two main components: the time interval between the beginning of symptoms and evaluation by the final assistant physician, and the interval between clinical evaluation and treatment itself. We were able to see that, in our population, the time before urological evaluation (presentation delay, PrD) was the dominant component of the delay in treatment. Elsewhere, there are examples that the responsibility for treatment delay may not lie solely with the emergency department staff, which can, point-of-fact, attain high diagnostic accuracy (6, 7, 21). Investigators from California noted that patients' transfers from primary and secondary to tertiary facilities was an independent risk factor for orchidectomy (22). In addition, an English study (15) revealed that caregivers waited in average 32 hours (range 3–240 hours) to bring a patient with acute scrotum to medical attention. This extended time was claimed to be responsible for their 45% salvage rate.

There is disagreement about the role of age in the prognosis of testicular torsion. One can find evidence attributing worse prognosis to younger (7–9, 23), older (10–12), as well as to patients at extremes of age, i.e., younger than 10 and older than 40 years (22). We, however, were unable to find any relationship between surgical testicular salvage and age, in both bivariate and logistic regression analysis. Therefore, among the subjects of our study, age did not influence surgical outcome.

In contrast, testicular rotation appears to play an important role in surgical outcome. The existence of some relationship between the amount of rotation, duration of ischemia and surgical outcome has been known for almost a century (2). There is experimental evidence (24) that 3 or 4 complete turns produce irreversible ischemic changes as early as 2 hours, whereas one turn can be tolerated for 12 hours, even though infarction inevitably occurs after 24 hours. Williamson asserted that there is no absolute time beyond which one can assume that infarction is inevitable (6). In his study, one organ was found necrotic after only 4 hours' ischemia, and 2 testicles were viable after 25 days of symptoms. In an analysis of 186 testicular torsion cases, Sessions (25) observed that salvaged testis twisted less than lost organs (360° vs. 540°). Despite the impressive number of cases, the data was not submitted to multivariate analysis, so the relative prognostic significance of time and magnitude of torsion was not investigated. A similar relationship was found by Cummings, that observed that mean rotation in salvaged cases was 412°, contrasting with 630° in non-viable testicles (11). From Kuwait, Al-Hunayan (26) was able to salvage 2 organs after 48 hours of ischemia, and noted that both had less one turn. Tryfonas (27), following 25 of his 75 treated cases, noticed that if rotation was greater than 360°, atrophy ensued after only 4 hours, in a manner similar to what occurred in all cases with ischemia lasting for more than 24 hours. He also noticed no later atrophy between 12 and 24 hours with rotations between 180° and 360°. In a study from China (4), median rotation in salvaged organs was significantly less than in lost organs: 360° (90°–540°) vs. 540° (90° – 960°) (r = −0.953, p = 0.011). These investigators called attention to 4 orchidectomy cases with less than 6 hours of symptoms, all of which had greater then 720° rotation. In our analysis, both presentation delay and rotation were significantly and independently associated with greater orchidectomy rates in iTT. Rotation had a crucial influence in surgical outcome, lowering the probability of orchidopexy even with very short treatment delays, in a dose–dependent manner.

The first consequence of our findings is that one has to deal with greater uncertainty in forecasting surgical testicular salvage in iTT, even with short treatment delays, since it is impossible to know the cord's rotation beforehand. Therefore, diagnostic imaging may not be affordable, for even minimal increases in treatment delay can cost dearly. One might, nevertheless, institute measures to reduce testicular ischemia, such as manual detorsion: manual detorsion can be successful even when converting a 4.0π radian to a 2.0π radian torsion, which could allow some testicular perfusion. Furthermore, there is some experimental evidence that ischemic injury can be reduced with local hypothermia (28, 29), a very simple measure. There is ongoing research on pharmacological therapy to reduce ischemic injury (30), but the possibility of serious adverse effects requires careful clinical consideration.

The second consequence is that surgical treatment should not be withheld with the argument that the testicle is all but lost, even in patients with long delays in presentation (>24 hours). Ten of our patients (7%) in such situation had their testicles surgically salvaged, including one with 100 hours of symptoms. One should keep in mind that patients with a single twist of the spermatic cord might preserve some testicular irrigation, and have a viable testis at surgical exploration. Moreover, the patient might be suffering from intermittent torsion, which explains finding a non-rotated testicle, even if typical symptoms are present.

This study has several drawbacks worth mentioning. The response variable of our analysis was surgical outcome, which is not the same as treatment outcome. Treatment outcomes will be most likely worse than surgical outcomes, since some surgically salvaged organs will eventually atrophy. Thus, even with our low surgical salvage rates, our results might have represented an optimistic scenario. Regarding our logistic model, we included rotation as a continuous variable, even though it was recorded at discrete intervals. On the other hand, the rotation of the cord must occur continuously: a 4.0π radian rotation has necessarily gone through lesser degrees, and, indeed, can be medically or spontaneously converted to a lesser degree. Furthermore, we also acknowledge the possibility of a non-linear effect of rotation on testicular perfusion, and recommend additional caution in the interpretation of the regression results. Lastly, we also did not have access to data on patient referrals and prior treatments, *e.g*., manual detorsion, so the use of treatment delay and its components as a surrogate for the duration of ischemia might be negatively biased.

## Conclusions

We have demonstrated, with non-parametric and multivariate logistic regression analysis, that both presentation delay, the dominant component of treatment delay for testicular torsion in our population, and testicular rotation were independently and inversely associated with surgical salvage in intravaginal testicular torsion. Most importantly, that testicular rotation exerts a multiplicative effect on ischemia time.

The exclusion of other causes of acute scrotum by imaging studies is currently accepted practice, provided surgery is not postponed. However, our results demonstrate that this might be a dangerous path, for there seems to be no safe period in intravaginal testicular torsion.

## Abbreviations

TrD: = Treatment Delay
PrD: = Presentation Delay
SurgD: = Surgical Delay
iTT: = Intravaginal Testicular Torsion
CI: = 0.95 Confidence Interval
OR: = Odds Ratio
ROR: = Relative Odds Ratio
IQR: = Interquartile Range
AIC: = Akaike's Information Criterion
